# Accurate Estimation of Heart and Respiration Rates Based on an Optical Fiber Sensor Using Adaptive Regulations and Statistical Classifications Spectrum Analysis

**DOI:** 10.3389/fdgth.2021.747460

**Published:** 2021-12-02

**Authors:** Rongjian Zhao, Lidong Du, Zhan Zhao, Xianxiang Chen, Jie Sun, Zhenzhen Man, Baogeng Cao, Zhen Fang

**Affiliations:** ^1^State Key Laboratory of Transducer Technology, Aerospace Information Research Institute, Chinese Academy of Sciences, Beijing, China; ^2^Zhongshan Zhongke Guangrun Technology Co., Ltd, Zhongshan, China; ^3^University of Chinese Academy of Sciences, Beijing, China; ^4^Naval Medical Center, Shanghai, China

**Keywords:** adaptive regulations, statistical classifications spectrum analysis, BCG, heart rate, respiration rate

## Abstract

The aim of this work is to present a method for accurately estimating heart and respiration rates under different actual conditions based on a mattress which was integrated with an optical fiber sensor. During the estimation, a ballistocardiogram (BCG) signal, which was obtained from the optical fiber sensor, was used for extracting the heart rate and the respiration rate. However, due to the detrimental effects of the differential detector, self-interference, and variation of installation status of the sensor, the ballistocardiogram (BCG) signal was difficult to detect. In order to resolve the potential concerns of individual differences and body interferences, adaptive regulations and statistical classifications spectrum analysis were used in this paper. Experiments were carried out to quantify heart and respiration rates of healthy volunteers under different breathing and posture conditions. From the experimental results, it could be concluded that (1) the heart rates of 40–150 beats per minute (bpm) and respiration rates of 10–20 breaths per minute (bpm) were measured for individual differences; (2) for the same individuals under four different posture contacts, the mean errors of heart rates were separately 1.60 ± 0.98 bpm, 1.94 ± 0.83 bpm, 1.24 ± 0.59 bpm, and 1.06 ± 0.62 bpm, in contrast, the mean errors of the polar beat device were 1.09 ± 0.96 bpm, 1.44 ± 0.99 bpm, and 1.78 ± 0.94 bpm. Furthermore, the experimental results were validated by conventional counterparts which used skin-contacting electrodes as their measurements. It was reported that the heart rate was 0.26 ± 2.80 bpm in 95% confidence intervals (± 1.96SD) in comparison with Philips sure-signs VM6 medical monitor, and the respiration rate was 0.41 ± 1.49 bpm in 95% confidence intervals (± 1.96SD) in comparison with ECG-derived respiratory (EDR) measurements for respiration rates. It was indicated that the developed system using adaptive regulations and statistical classifications spectrum analysis performed better and could easily be used under complex environments.

## Introduction

In order to address the potential concerns of chronic diseases and sub-health status, the development of remote, intimate, and immediate medical treatments with extensive mobile network coverage is really significant. Especially, with the coming of aged societies, the urgent need of remote, miniaturized, and portable healthcare equipment is imperative for health monitoring, adjuvant treatments, and rehabilitation exercises to every user (1–3). Many physiological parameters can be obtained by this equipment. The cardiopulmonary function which means measuring the heart rate (HR) and the respiration rate (RR) is the most important function of this device. The heart rate, which is represented by the number of contractions of the heart per minute, is closely correlated with various heart diseases, such as sinus tachycardia, heart arrhythmia, and premature ventricular contraction. As for the respiration rate, which is a parameter referring to the number of breaths per minute, it can be used as an indicator for various abnormal physical conditions such as asthma, anxiety, pneumonia, and drug abuse.

Traditional approaches of monitoring heart and respiration rates include (1) using an electrocardiogram (ECG) (4, 5) to record electrical activities of the heart over a period of time by the close connection between the electrodes and the skin of subjects, (2) using photoplethysmography (PPG) (6–8) to reflect the cardiac-induced changes in tissue blood volumes according to the measured results of probes which are clamped to fingers, and (3) using polysomnography (PSG) (9) to comprehensively record biophysiological changes of subjects while sleeping. However, these methods are not suitable for long-term monitoring due to their inconvenience and the discomfort they bring to the subjects. Recently, as an alternative approach of collecting physiological parameters without close contact with subjects, the ballistocardiogram (BCG) has been widely researched (10–13). More specifically, BCG can be used to measuring the vibrations from the head to the soles of the subjects due to blood pumping. The current studies of extracting BCG signals have been focused on the developments of the approaches and the platforms which include electromagnetic wave scanning reflectors (14), piezoelectric films (EMFI) (15), seismic detectors (16), optical fibers (17, 18), and so on. Among these approaches and platforms, electromagnetic wave scanning reflection is difficult to implement because it can be easily influenced by external environmental variations and it requires complicated hardware. Piezoelectric films also have their obvious disadvantages such as poor sensitivities and high production costs. Furthermore, piezoelectric films require multi-level amplifications which increase system complexity and production costs drastically. Seismometers are relatively expensive and insensitive to respiration. Fortunately, as a non-inductive equipment, optical fibers can could be used to obtain physiological parameters with high reliability, durability, and low cost, especially in harsh environments. In this paper, thus, an optical fiber-based mattress system was developed and could be used not only for professional medical environments but also within households.

However, the ballistocardiogram (BCG) signal based on a fiber sensor is difficult to detect due to the detrimental effects from the different subjects, self-interference, different installation status, and so on (19, 20). Firstly, the signals received by the optical fibers are very weak, thus the corresponding extraction of physiological parameters is challenged (9). Secondly, some physiological movements, such as snoring in sleeping, can deform the waveforms of BCG. Furthermore, the waveforms of BCG would vary significantly from different subjects, and even for the same subject but in different recording periods (21). Finally, the variation of installation status of the sensor has a significant impact on the BCG signal. So, in this study, a specially designed detection method was developed in order to accurately extract heart and respiration rates from BCG signals on the basis of a fiber sensor. First of all, the optical fiber system was designed with an adjustable transmitter driving circuit and a trans-impedance amplifier. Moreover, thanks to the real-time feedback voltages from an adaptive feedback algorithm used in this study, the individual differences could be adjusted in time. Furthermore, statistical classifications based on the analysis of signal spectra were conducted to locate fundamental and harmonic frequencies, which was beneficial for the extraction of heart and respiration rates.

The main work of this study is organized as follows: Introduction summarizes the progress and problems in measuring BCG; Methods introduces the acquisition system on the basis of an optical fiber mattress and provides the developed extraction method of BCG signals; Results and discussion concludes the measurements of heart and respiration rates on the basis of the developed system; and Conclusions is the conclusion.

## Methods

### System Setup

**Figure 1:**
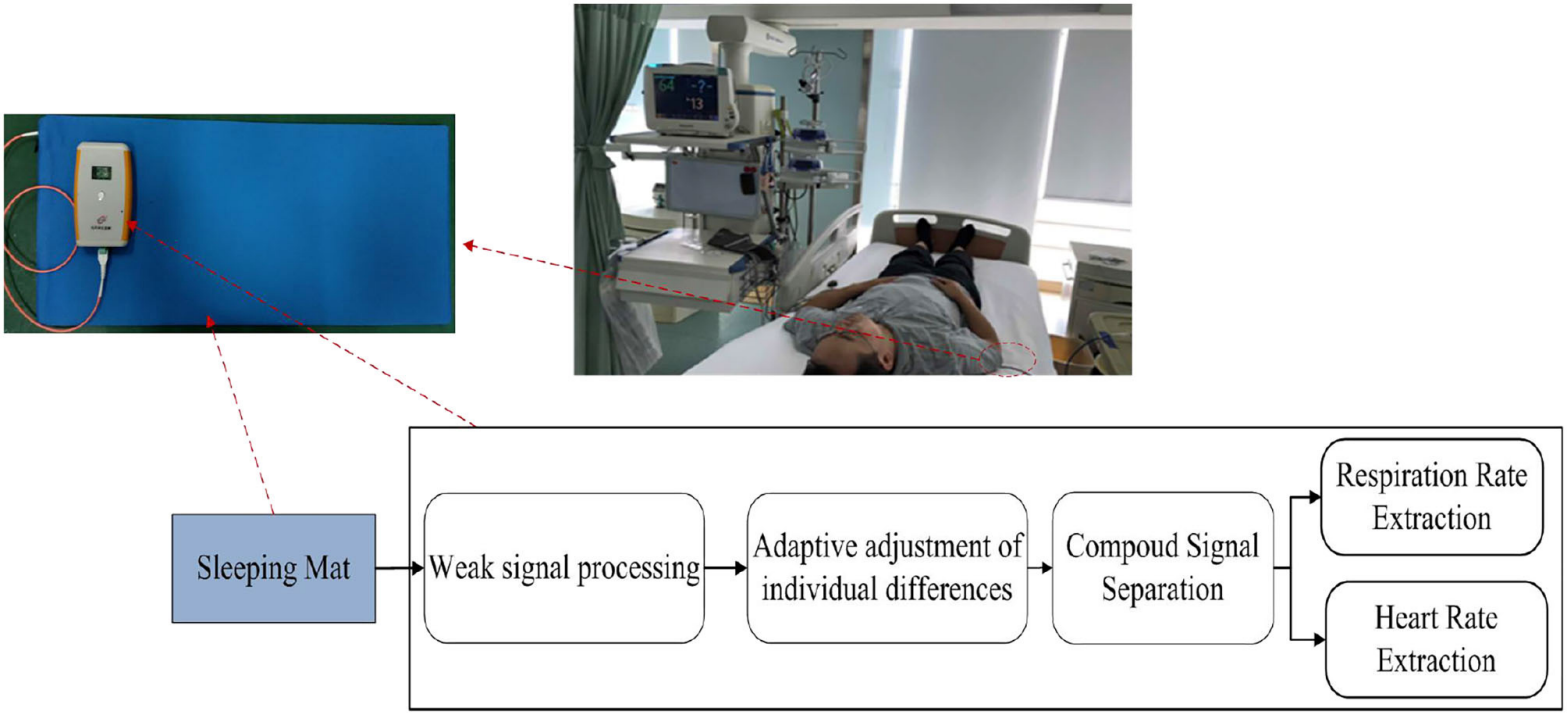
Block diagram of the BCG signal acquisition system.

**Figure 2:**
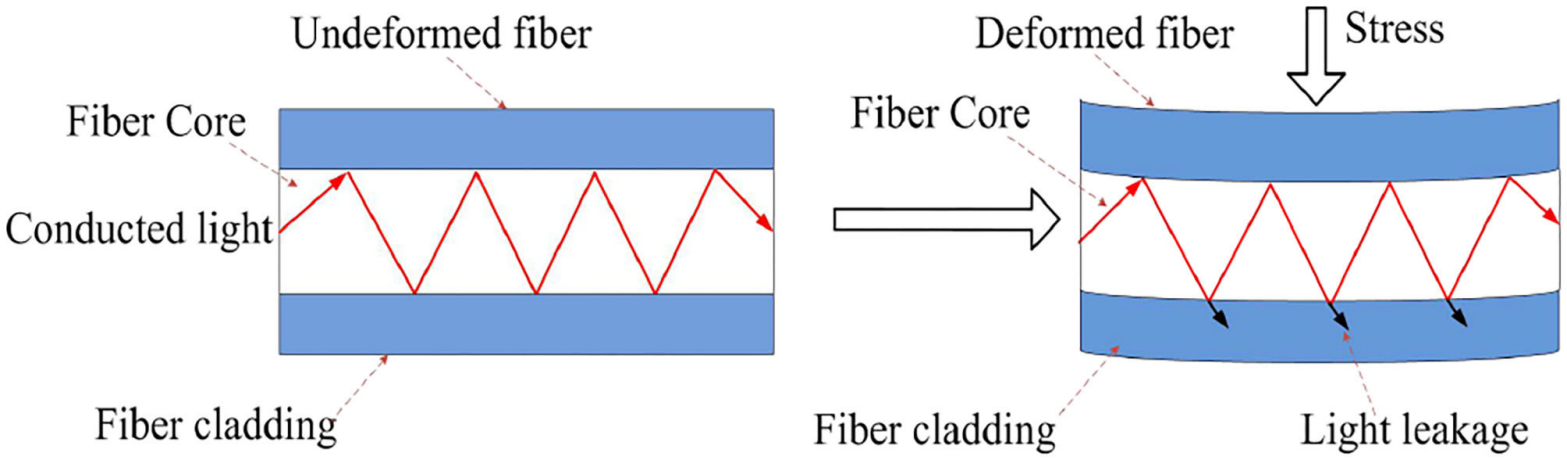
Optical conduction loss caused by mechanical deformation of micro-bending fiber under the action of stress.

The block diagram of the BCG signal acquisition system is shown in Figure 1. A mattress, in which an optical fiber was embedded, was placed on a bed. After a subject laid on the mattress, the micro-bending changed pre-stress and was fixed. Then, the heart vibration of the subject brought additional stress to the mattress which led to fiber deformation and finally changed the light transmission (Figure 2). Subsequently, the decaying light signals were converted into current signals by the light detector which was coupled with the received connector. Next, current signals were amplified and converted to voltage counterparts, and adaptive adjustments of individual differences(see Adaptive Adjustment of Individual Differences) were conducted to confine the measured signals within an optimal range. Finally, signal processing was conducted to extract key physiological parameters including heart and respiration rates.

### Structure of the Optical Fiber-Embedded Mattress

**Figure 3:**
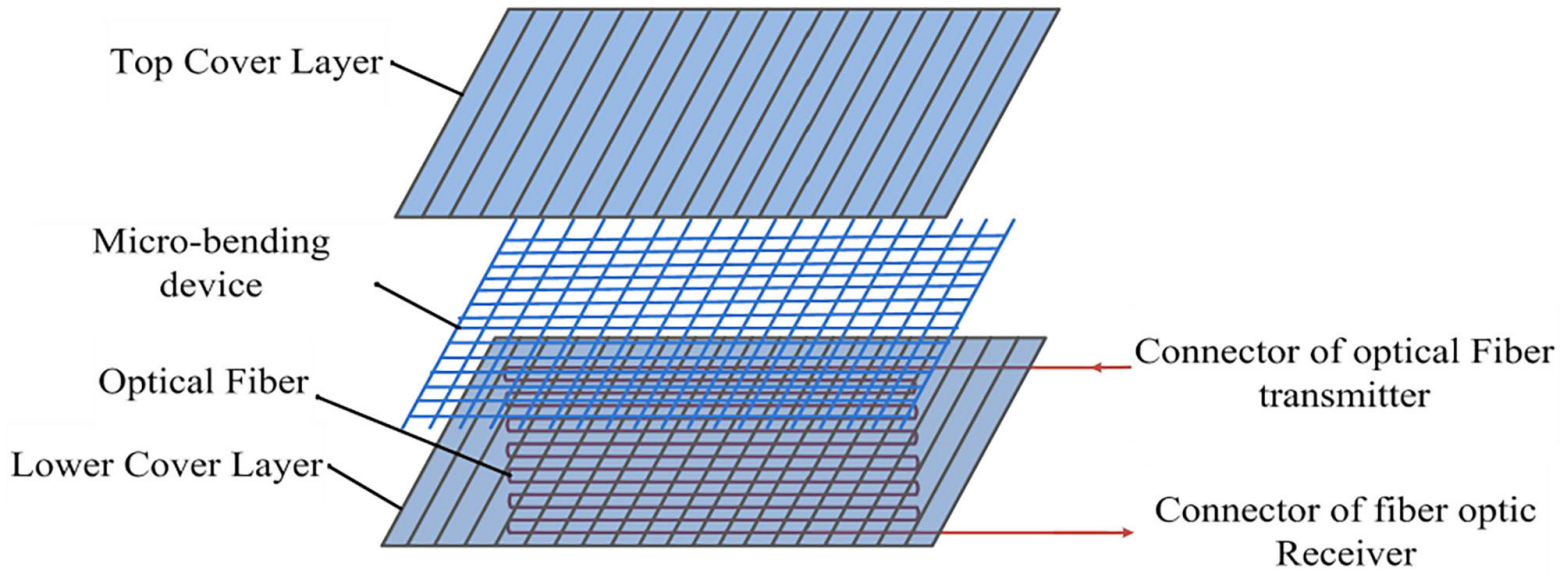
Internal structure of the optical fiber mattress.

The structure of the optical fiber mattress is shown in Figure 3. It mainly consisted of a sensitive section and a collection of transmit and receive connectors. The sensitive section included gradient multimode fiber (MMF), a micro-bending device, and the upper and lower covering plastic materials (22, 23). Where the MMF was located in the center of the entire structure, close to the micro-bender, the upper and lower two layers of soft covering material were used to clamp the MMF and micro-bender in the middle, forming a sandwich structure. The soft material, which covered above and below the sandwich structure, could protect the fiber well and improve the reliability and stability of the mattress. At the same time, in order to ensure the high sensitivity of the fiber mattress, it was required that the fiber optics were evenly distributed. In order to meet the above requirements, the design used a serpentine back-fold ingress sloth to distribute the fiber optic sensor evenly in the middle of the mattress, while using MMF to improve optocoupled efficiency and enhance detection sensitivity.

First of all, the design of the optical fiber mattress was based on a previously reported theoretical model (24). The change of light transmission in response to an applied stress is defined as:


(1) $$\begin{array}{l} \Delta C=\left(\frac{\Delta C}{\Delta X}\right)▪\Delta F▪\Delta E \end{array}$$

Where Δ*F* is the applied stress, Δ*C* is the coefficient variation representing the change of light transmission, Δ*X* is the change of small deformation, ΔE is the original environment change that is connected with some parameters which include the pressure coefficient (*K*), the cross-sectional area (*A*), the Young's modulus (*Y*), and the mattress length (*L*). Δ*E* is shown as follows:


(2) $$\begin{array}{l} \Delta E={\left(K+\frac{A▪Y}{L}\right)}^{-1} \end{array}$$

Where *K* is the force constant of the fiber, which is associated with the fiber diameter (*d*) and the number of bends (γ) as follows:


(3) $$\begin{array}{l} K=3▪\pi ▪Y▪d^{4}▪\gamma \end{array}$$

In order to achieve the highest sensitivity of the fiber mattress, the above parameters were carefully selected. To be more specific, the fiber was evenly distributed in the middle layer of the mattress and closely followed the bend line in a pattern of serpentine return. Furthermore, the MMF (composed of silica) was used to enhance the efficiency of optical coupling because its core diameter is much larger than single-mode fibers. The micro-bending device was also added to increase the deformations of the fiber. Finally, the plastic cover was painted to protect the optical fiber and improve the reliability and stability of the mattress.

### Weak Signal Processing

**Figure 4:**
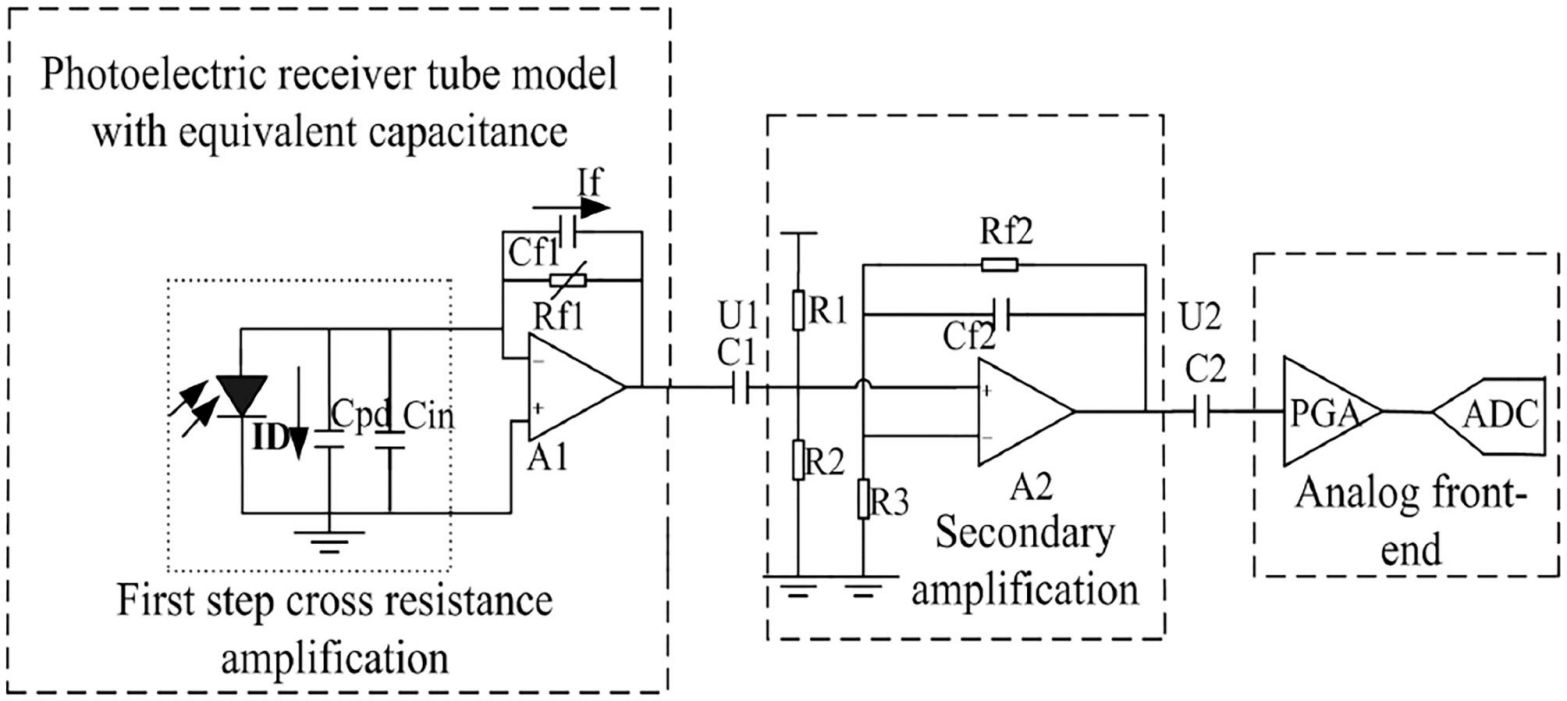
Preprocessed circuit including three-stage amplification.

The key issue of obtaining the electrical signals from the optical fiber mattress was that the signals had small amplitudes. This paper used the following methods to solve this issue. Firstly, the weak current signal was translated into a voltage signal with amplification by a trans-impedance amplifier. For weak signal processing, current and voltage noises of the system must be kept at a low level. Secondly, the optical fiber transmitter and receiver were placed within a metal shield and shielding rings were included in the transmitting and receiving circuits. The wavelength of the fiber optic sensor was 900–1,650 nm, with a typical value of 1,310 nm and a saturated power of 10 mW. It was shown that the current level of the detector was at the nA level, and the signal amplitude after the first-step cross-resistance amplification was approximately 40 mV. In practice, the feedback resistor brought more noise into the circuit as well, although increasing the feedback resistor improved the magnification ratio. As a result, the front two levels of the amplification circuits mainly consisted of the OPA656 (TI) amplifier, and the third-level amplification adopted an analog front-end as a 16-bit high-precision sigma delta AD of an ADS1115 (TI) sampling circuit to realize weak signal processing (see Figure 4).

### Adaptive Adjustment of Individual Differences

When the fiber mattress was under force, the amplitudes of the received signals varied significantly from subject to subject. In order to address this problem, an adaptive feedback algorithm was used to automatically adjust the controlling voltages of the transmitter driving circuit and the magnification of the amplification circuit. At the same time, a feedback loop was also proposed for the acquisition and control of the optical fiber which could further eliminate individual differences dynamically.

**Figure 5:**
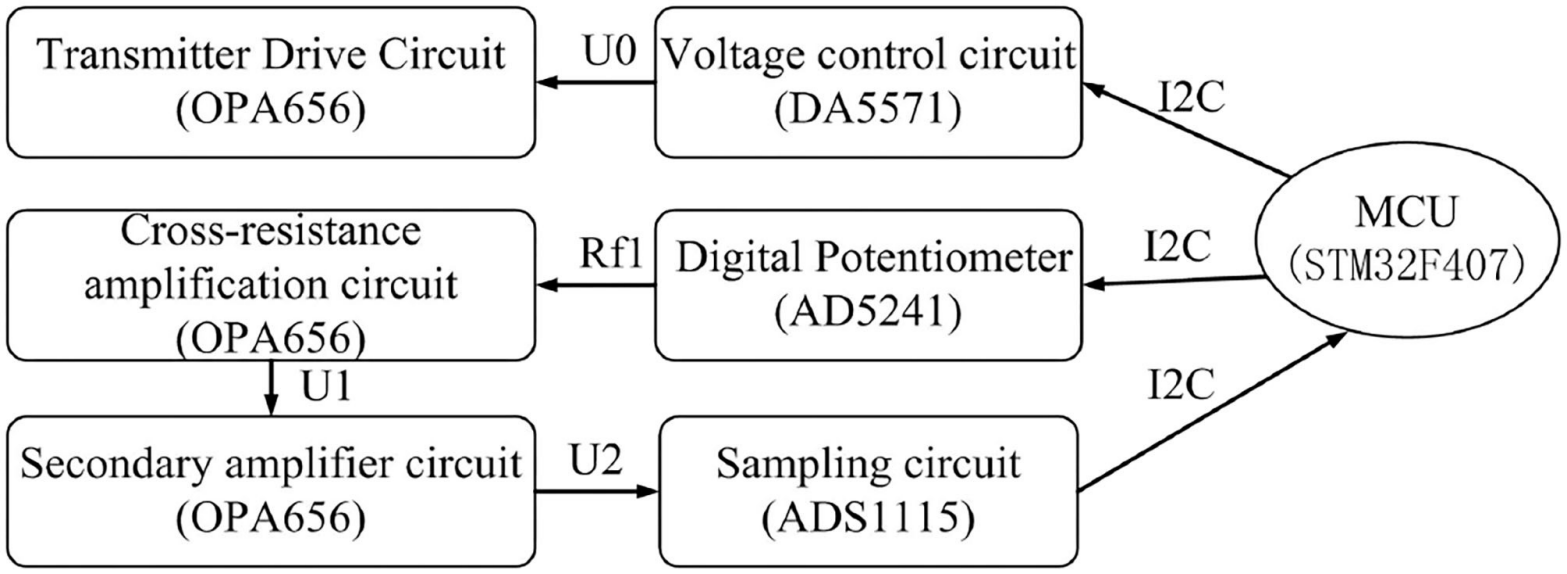
The feedback loop of the fiber acquisition control.

The workflow of the feedback acquisition and the control flow of optical fibers are described as follows (see Figure 5). After a subject laid on the fiber mattress, the initial value of the transmitter driving voltage U0 and the magnification (Rf1 and U2/U1) of the amplification circuit were fixed. Then the main control chip MCU received an AC signal which was from the sampling and was used to judge whether the amplitude of the signal was within an appropriate range or not. If the single was within the appropriate amplitude, no adjustment would be conducted. Otherwise, the output voltage of the control circuit (transmitter drive voltage U0) would be changed to adjust the magnification of the amplifier. Finally, it formed a collection control feedback loop to confine the output voltage in the best detection range.

In the application, the master chip STM32F407 received the AC signal of the ADS1115 via I2C, and then, according to the size of the DC component of the AC signal, the drive voltage U0 of the fiber transmitter was controlled by the DA5571 module and the variable resistance of the digital interline AD5241 to achieve the cross-block amplifier magnification control, and finally form a receiver control ring to achieve the best output.

### Compound Signal Separation

**Figure 6:**
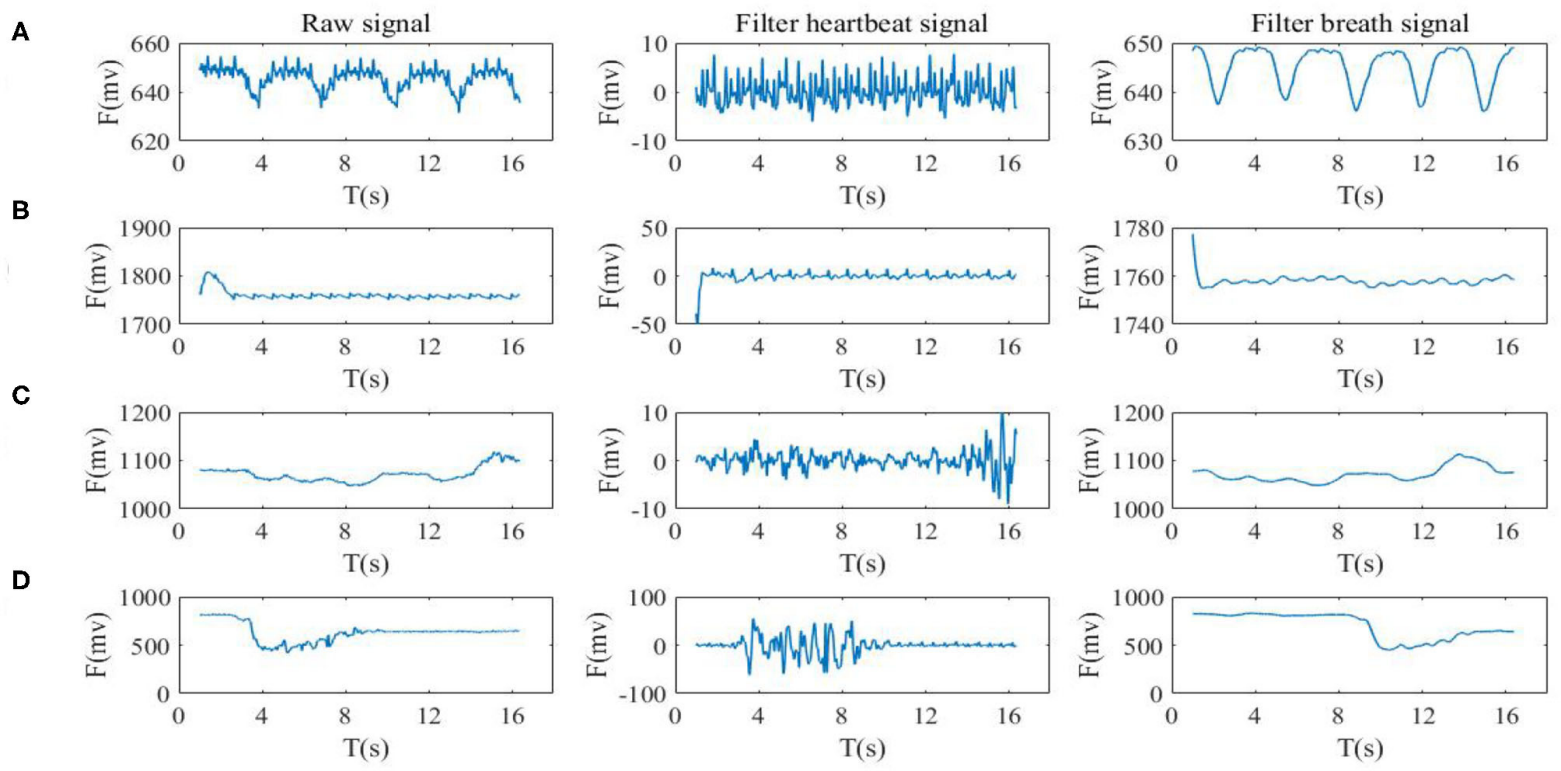
Signal separation of BCG. The left side is the original signal of the sensor, and the right side is a pre-treated BCG signal according to the band filter **(A)** static state waveform, **(B)** the holding breath, **(C)** slow respiration, and **(D)** the movement state.

The waveforms, which were pre-processed after being adaptively adjusted, were inevitably contained by the components of breath, muscle fibrillation, and heart pumping. Thus, it was difficult to directly extract heart and respiration rates from the waveforms. As the amplitudes and frequencies of respiration were different from heartbeat, a 9–25 Hz band filter was used to separate heart beat waveforms and a 0.6–10 Hz low-pass half-band filter was used to extract the breath waveforms (see Figure 6).

The band filter had the effect of selecting useful frequencies to pass through and suppressing useless frequencies. After setting the vibration signal to *x*(*n*), then the output signal through the FIR is:


(4) $$\begin{array}{l} {\text{y}}_{\text{f}}\left(\text{n}\right)=\overset{\text{N}}{\underset{\text{i}=0}{\sum }}{\text{h}}_{\text{i}}\text{x}\left[\text{n}-\text{i}\right] \end{array}$$

Where h_i_ is the system function of the filter, expressed as a finite pulse response. The coefficient of b_j_ for the filter is as shown as


(5) $$h_{i}=\sum_{\text{j=0}}^{\text{N}}{\text{b}}_{\text{j}}\delta [\text{i-j}]$$

The corresponding Z transformation is represented as:


(6) $$\text{H}\left(\text{z}\right)=\sum_{\text{n}=-\text{∞}}^{+\text{∞}}{\text{h}}_{\text{n}}{\text{z}}^{-\text{n}}$$

**Figure 7:**
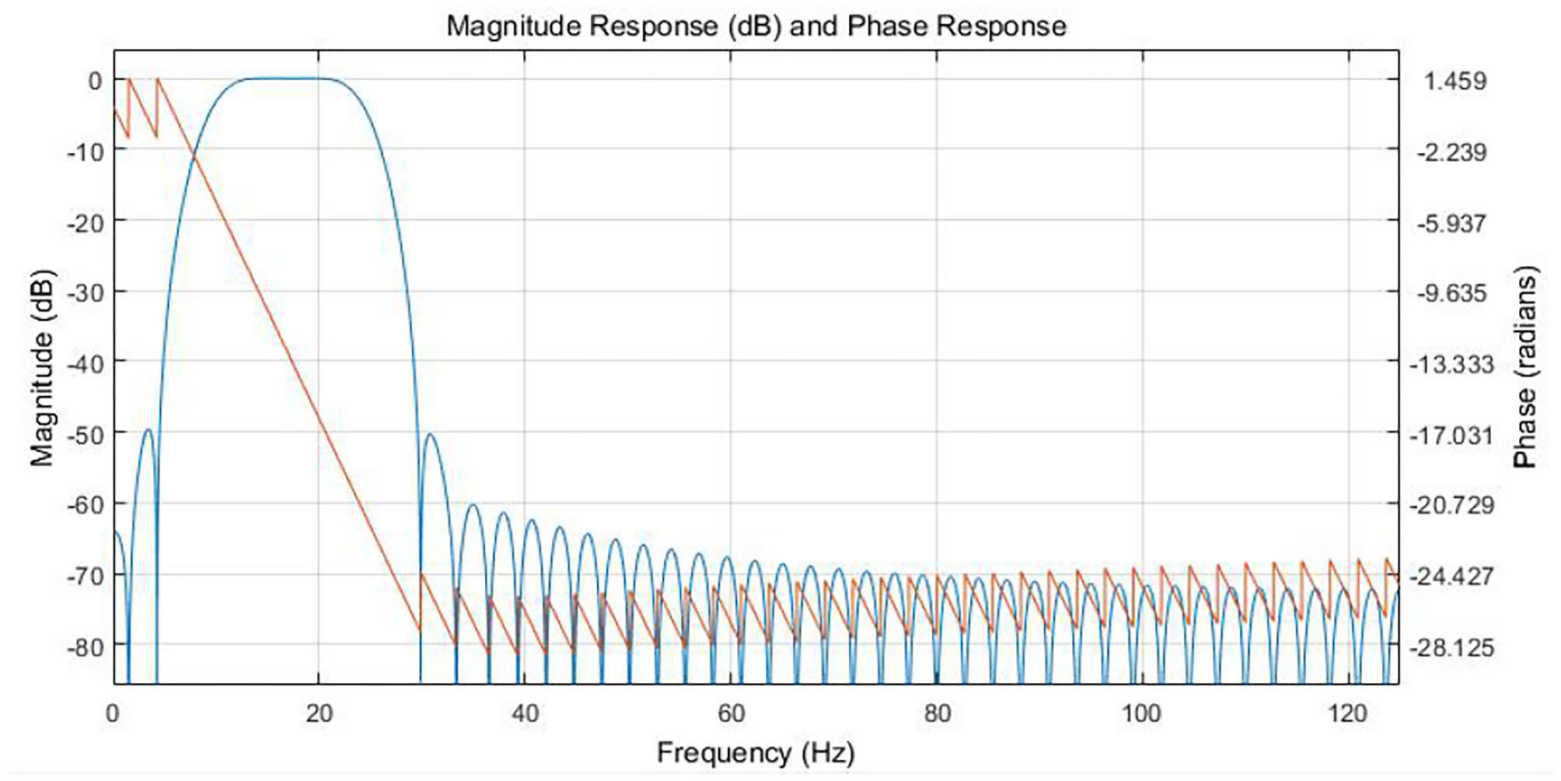
Response relationship between amplitude frequency and phase frequency of 9-25 Hz six-order FIR Filter.

Based on the frequency band characteristics of the vibration signal, the FIR filter parameters were of 90 orders and the band pass filter with a band range of 9–25 Hz was designed. The band and phase frequency characteristics are shown in Figure 7.

### Respiration Rate Extraction

The respiration waveforms were extracted by the aforementioned methods (see Figure 6).

**Figure 8:**
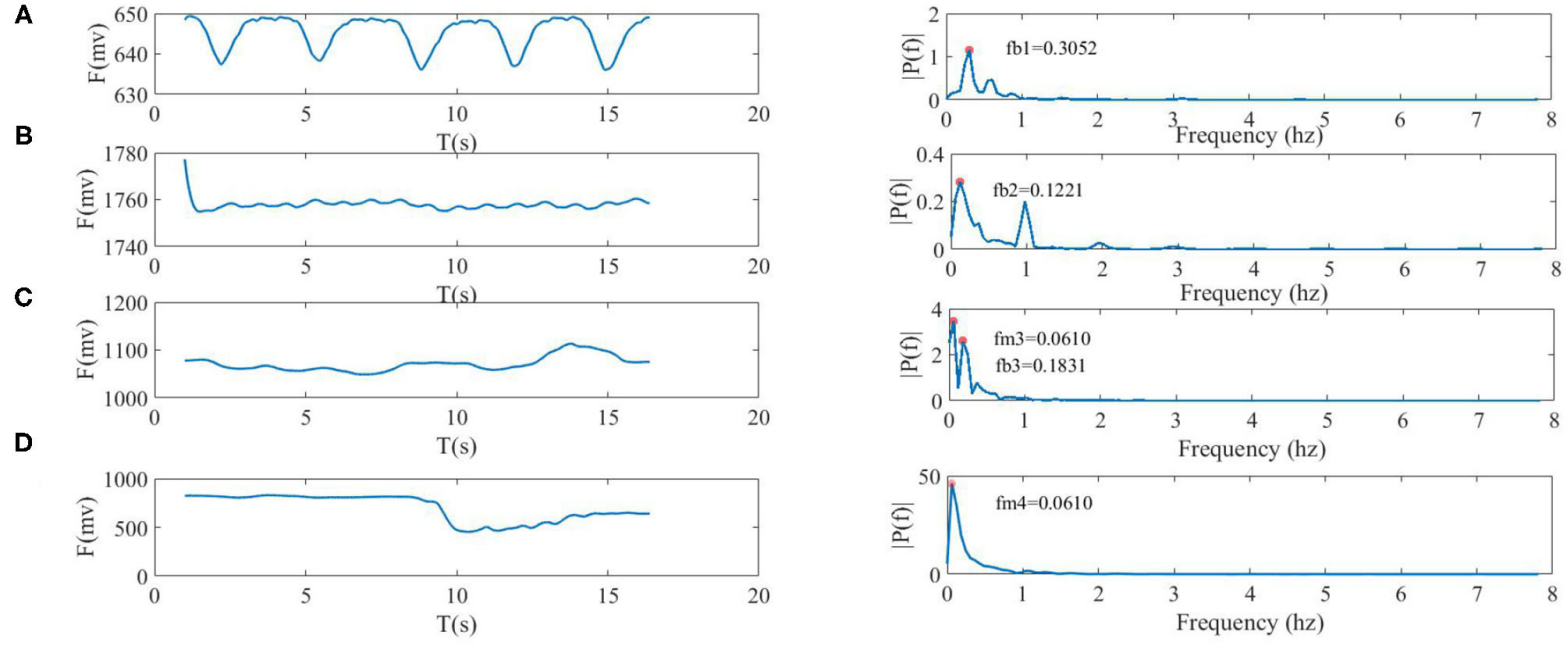
Four types of breath waveforms (the left side) and spectra (right side), where fb1, fb2, and fb3 were frequencies of respiration, and fm3 and fm4 were frequencies of movement. **(A)** static state, fb10 = 0.3052 Hz, **(B)** the held breath state, fb20 = 0.1221 Hz, **(C)** slow breath was accompanied by body movement which included fb30 = 0.1831 Hz and fm3 = 0.0610 Hz, and **(D)** the movement state only included fm40 = 0.0610 Hz of moved frequency.

The detailed procedures for calculating respiration rates are shown as follows:

1. Pre-processing the BCG signals by a comb filter;
2. Conducting FFT processing;
3. Finding the maximum spectrum point in the range of 0.1–0.5 Hz (see Figure 8) with four states (e.g., static state, holding breath, slow breath, and movement);
4. Calculating the respiration rates based on formula 4 at the corresponding frequency point, where *f*_max_ is the peak frequency within 0.1–0.5 Hz, for example, *f*_max_ could be fb1, fb2, or fb3, and *R*_*r*_was expressed as the respiration rate;
5. Refreshing the data once every 128 points, and then returning to step (1).

(7) $$\begin{array}{l} R_{r}=f_{\max}▪60 \end{array}$$

### Heart Rate Extraction

**Figure 9:**
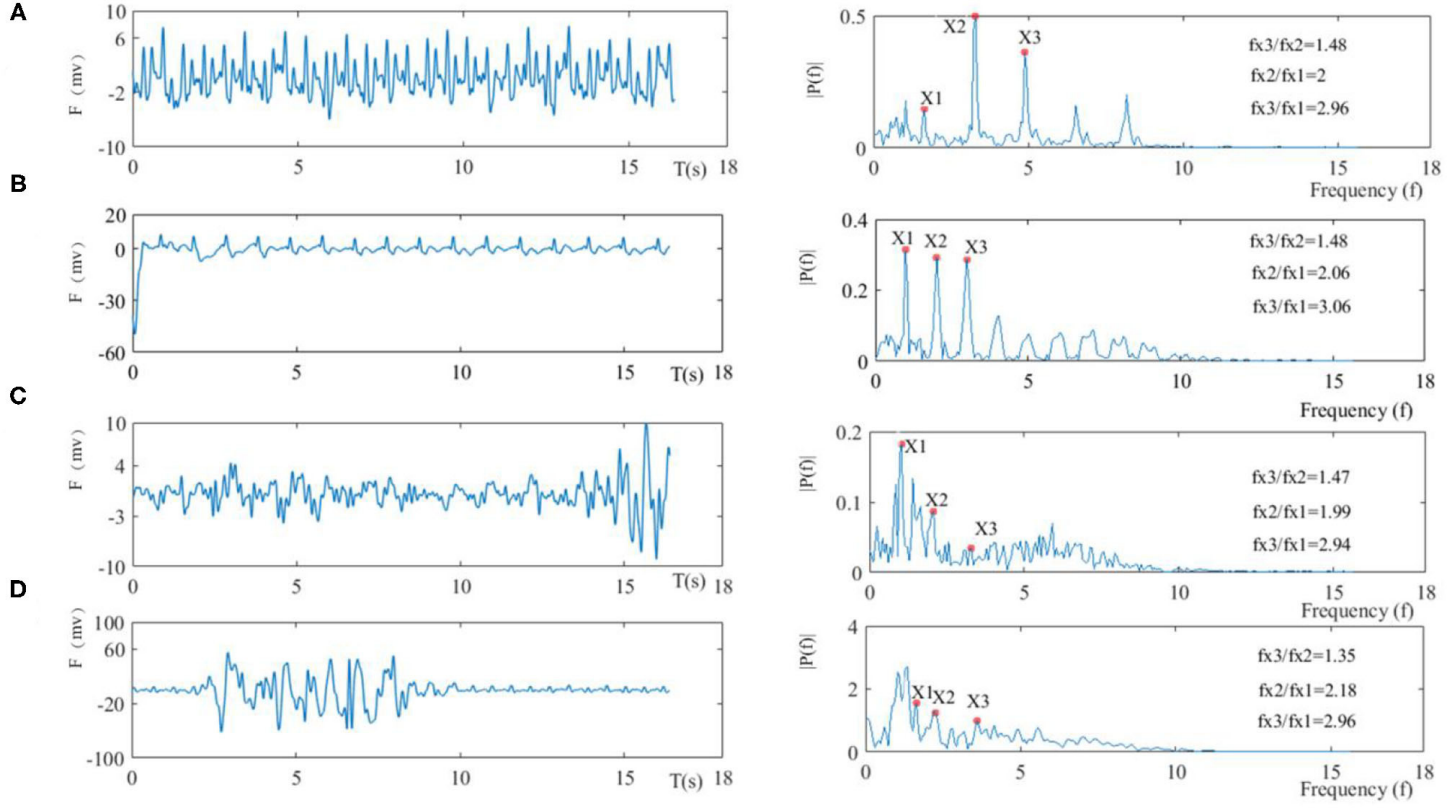
Four types of BCG waveforms (the left side) and spectra (right side), in which the fundamental frequency is X1, the second harmonic is X2, and the third harmonic is X3, **(A)** in the normal resting state, **(B)** in the holding breath, **(C)** in the case of slow respiration, and **(D)** in the moving state.

The waveform of the heart beat was extracted by the aforementioned methods which are shown in Figure 6. Firstly, before presenting the algorithm, the morphologies of the heart beat waveform were discussed. As shown in Figure 9A, individual peaks of the heart beat were submerged under normal and slow breath states (Figure 9C). Conversely, when the subject held their breath (see Figure 9B), there were more clear and prominent peaks on the heart beat waveform after a slight jittering which happened before entering this state. These results indicated that breath had varying degrees of interference with the heart beat waveform. In addition to the breath interference, motion disturbances such as physical movements also produced inconspicuous peaks in the heart beat waveforms. Figure 9D shows that motions and heart beats were superimposed which led to serious interference in the waveform morphologies. Therefore, the algorithm of peak detection was not effective in this situation which means it was difficult to estimate the waveform morphologies.

Secondly, the spectrum of the heart beat waveform was introduced, and the base frequency was the frequency component with the highest magnitude peak in the spectrum around the lowest frequency, which was consistent with heart beat frequencies within 0.6–3 Hz in the heart beat waveform. Harmonics with frequencies which were whole-number multiples of the base frequencies were composed of BCG signal components. Moreover, the relationship of base frequencies and harmonics satisfied Formula 8, where fx1 is the base frequency, fx2 is the second harmonics, and fx3 is the third harmonics.


(8) $$\begin{array}{l} 6▪f_{x1}=3▪f_{x2}=2▪f_{x3} \end{array}$$

Four types of heart beat waveforms (left side) and spectra (right side) are shown in Figure 9. In the resting state, it can be seen that the amplitudes of the second and third harmonics were prominent, but the amplitude of the base frequency was low due to respiratory interference. Still, the relationship between two of the three frequency points was close to Formula 8 with the detailed relationship shown in Figure 9A. The held breath state (see Figure 9B) had an obvious base frequency compared to the normal resting state besides prominent harmonics and a good frequency relationship, therefore, base and harmonics frequencies were judged based on the maximum amplitudes. The slow breath state (see Figure 9C) suggested that amplitudes of harmonics were relatively low due to breath interference, and thus base and harmonics frequencies were not judged by only amplitudes since peaks of other interferences were more prominent, while the relationship between three frequency points was still close to the relationship in Formula 9. In the moving state (see Figure 9D), it was shown that the amplitudes of base frequency and harmonics were not obvious.

### Statistical Classifications Spectrum Analysis

According to the above discussion, the calculation of heart rate can be converted into the extraction of the frequency of the base and harmonic wave in the spectrum. In particular, when the relationship of base frequency and harmonics was close to Formula 8, the ratio of signal to noise was higher and the heart rate could be judged by only finding the maximum amplitudes of three frequencies (*fx*_1_, *fx*_2_, *fx*_3_). Heart rate extraction was very difficult when the relationship between the base frequency and harmonics was not obvious. In this way, statistical classification and optimization must be further adopted to extract the base frequency and harmonics.

The detailed flow chart of calculating heart rates is shown as follows:

#### FFT

The frequency resolution of filtering BCG signals was 1 Hz under an AD sampling rate of 250 Hz. In order to accurately extract heart rates, a down sampling operation of 16 times was used before FFT and thus the frequency resolution reached 0.015 Hz to meet the heart rate extraction requirements.

#### Location of Frequency Points

Combined with the characteristics of the signal's spectrum, the relationship between the base frequency and harmonics should be as consistent as possible with Formula 8. In other words, the ratio of frequency should meet Formula 9, where φ was a suitable threshold where the right heart rate could be found. In most cases, the appropriate frequency points can be selected based on the maximum spectral amplitudes that simultaneously satisfy the following formula.


(9) $$\begin{array}{l} 1.5-\varphi <\frac{f_{x3}}{f_{x2}}<1.5+\varphi \\ 2.0-\varphi <\frac{f_{x2}}{f_{x1}}<2.0+\varphi \\ 3.0-\varphi <\frac{f_{x3}}{f_{x1}}<3.0+\varphi \end{array}$$

Then, after each spectral transformation, the frequency value of *f*_*x*1_ was found. Identified heart beats HR (in Formula 10) were stored.


(10) $$\begin{array}{l} HR=f_{x1}▪60 \end{array}$$

**Figure 10:**
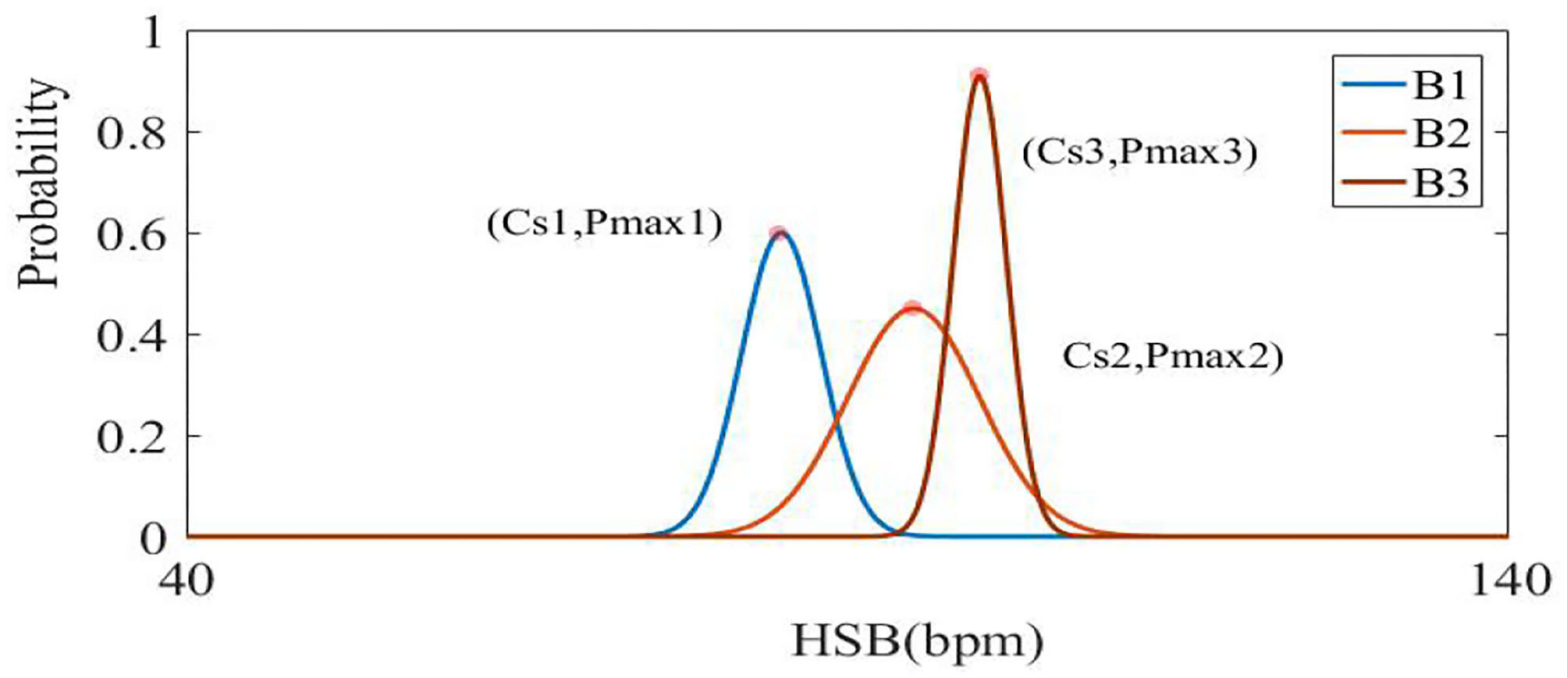
The visualization of B1, B2, and B3 curves represents each update process of statistical probability distribution (from distribution B1–B2, B2–B3). The horizontal axis represents the division of the sub-partition, and the ordinate represents the probability value in a certain interval.

HR (heart rate in Formula 10) was calculated to fall into a sub-particle of the heart beat (HSB) at sufficient intervals, and the probability distribution under this HSB is shown in Figure 10. Specifically, HSBs were set into 44 categories between 40 bpm and 140 bpm, where the category was marked as *Cj*, and the median of the heart beat under each category was marked as *HRj*. When HR was refreshed, the probability was redistributed from B1 to B2, and B2 to B3. The category *Cs* with the max probability (Pmax) was found, and then the median *HRs* under this category were taken (shown in Formula 11).


(11) $$\begin{array}{l} \{\begin{array}{c} Cs=\arg\max\left\{P▪\left(Cf|HR\right)\right\}\cdots j\in \left[1,44\right]\\ HRs=median\left(Cs\right) \end{array} \end{array}$$

(12) $$\begin{array}{l} probability=probability▪0.75\cdots P\;\max>TH1 \end{array}$$

When Pmax reached a certain value of TH1, it reduced the distribution height of the curve to realize the dynamic refreshment (see Formula 12). In our system, TH1 was set as 0.8.

#### Optimization

When the signal was disturbed by movement and respiration, the correlation between the fundamental frequency and the harmonic was not obvious. In order to overcome this problem, whole frequencies were first found in the spectrum, and then the statistical value *HRs* were combined to find the best base frequency and harmonics.

Based on all the peaks of the spectrum(*x*_1_, *x*_2_, …, *x*_*n*_), the frequency of 0.666–9 Hz was divided into three categories. According to the relationship between the base frequencies and harmonics where M1, M2, and M3 represent three intervals of 0.666–3 Hz, 1.332–6 Hz, and 1.998–9 Hz, respectively. A sequence of (*x*_1_, *x*_2_, …, *x*_*k*_) can be extracted under the limit condition of three-time frequency (in Formula 13), where the MM1, MM2, and MM3 have K sequences which respectively contain sets of the fundamental frequency, second harmonics, and third harmonics. The statistical heart rate Ha was considered as a reference for differences between *HRf* (final heart rate) and *HRs*. Since the sudden change was no more than 100 ms during the period of heart rates, we set τ <100 ms (25) to prevent abnormal errors.


(13) $$\begin{array}{l} \{\begin{array}{l} HRf=60▪f_{r1}\;|HRf-HRs|<100ms\\ HRf=HRf\;otherwise \end{array} \end{array}$$

Finally, the best set of frequency points (*f*_*r*1_, *f*_*r*2_, *f*_*r*3_) was found from k sequences to calculate the final heart rates *HRf* (Formula 13).

### Experimental Design

In order to evaluate the method for improving heart rate and respiration rate estimation, some experiments were designed by integrating an optical fiber sensor into a mattress under a variety of conditions. The experiments were conducted in accordance with the principles embodied in the Declaration of Helsinki and in accordance with local ethical requirements.

#### Participants

A total of 26 male and 14 female healthy volunteers aged between 20 and 60 years old who weighed between 40 and 80 kg participated in the experiment.

The first part of the test was to observe the effects of a single condition with other specific conditions under control.

#### Procedure

(A) Firstly, for individual subjects, we calculated the voltage amplitude of the subject while they lay on the mattress. Next, the BCG waveforms were affected by a variety of conditions. The interference was more pronounced because the vibrations of the heart beats were weaker than thoracic movements of breathing. Then the algorithm of extracting heart rates were mainly verified, and ECG was used for comparison. (B) Heart rates were compared with each other under different breathing conditions, such as apnea (simulate breath holding state), normal breathing, and slow breathing conditions. (C) Then the performance of the optical fiber mattress in different moving states (e.g., slight and large moves) was tested. (D) Experiments verified that the system can measure heart rate in different posture states.

The second part of the test was designed to evaluate the performance of the developed system in practical environments.

#### Procedure

(E) In particular, there are many things that we could not predict at night, for example, wide-range changes of heart and respiration rates. In order to validate whether the system can keep up with the changes of heart and respiration rates, a 5-h nightly experiment was conducted in comparison to commercial polar beats of H10 medical equipment and electrocardiogram-derived respiratory measurements. (F) The test was applied to verify heart rates at different intensities by exercises and respiration rates using controlling breath stages. These results were compared with the Philips sure-signs VM6 Medical Monitor at the Naval General Hospital and Tiantan Hospital (Beijing, China).

## Results and Discussion

### Verification of Individual Differences

The participants made contact with the fiber mattress in three different postures, and their signal amplitudes were stored [unit: voltage (V)]. The experimental results showed that original signals were in the range of 1–2 v due to individual differences (see Table 1). Therefore, the system can be adapted to the subject's weight in the range of 40–80 kg.

**Table 1:** Individual differences.

**Range of weight (kg)**	**Back (V)**	**Left (V)**	**Right (V)**
40–50 kg	1.76–1.81	1.75–1.76	1.66–1.70
50–60 kg	1.56–1.58	1.59–1.60	1.1–1.2
60–70 kg	1.43–1.45 V	1.36–1.39	1.19–1.2
70–80 kg	1.77–1.83 V	1.05–1.06	1.65–1.70

### Verification of Heart Rates Under Different Breathing Conditions

**Figure 11:**
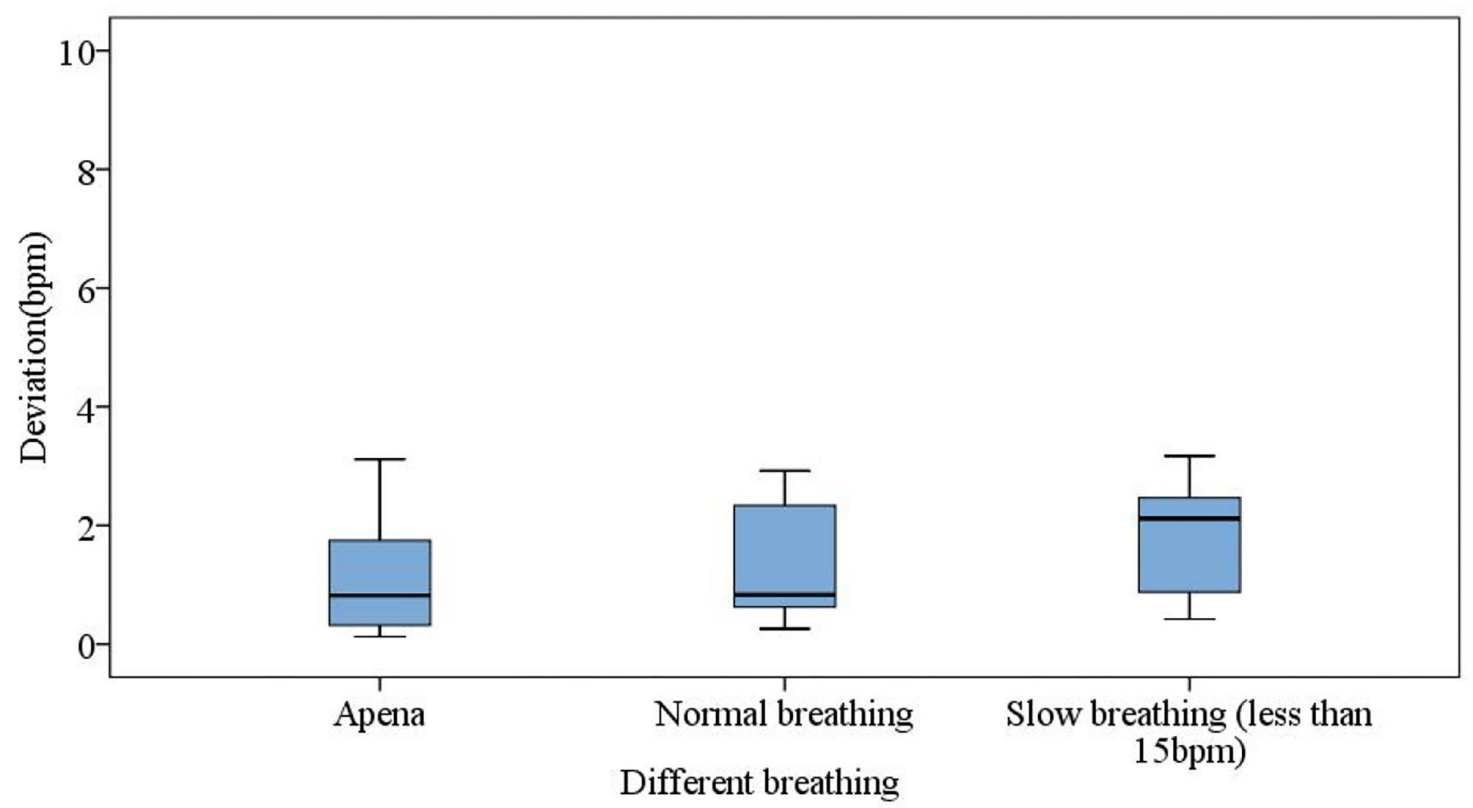
Different breathing states for apnea (breath holding), normal respiration, and slow respiration.

The following three kinds of states were measured. It is shown in Figure 11 that the averaged error of measuring heart rates in apnea (breath holding) was 1.09 bpm, and most of the deviations were within 2 bpm. At the same time, the mean error of normal breath was 1.44 bpm with most of the deviations within 2.5 bpm. Under the condition of slow breathing, the mean error was 1.78 bpm. As a result, the measured heart rates under normal respirations and apnea were more consistent with the standard values, while slow breathing inevitably increased thoracic amplitudes, which affected the calculation of heart rates with larger mean errors.

### Measurement Errors Under Different Postures

**Figure 12:**
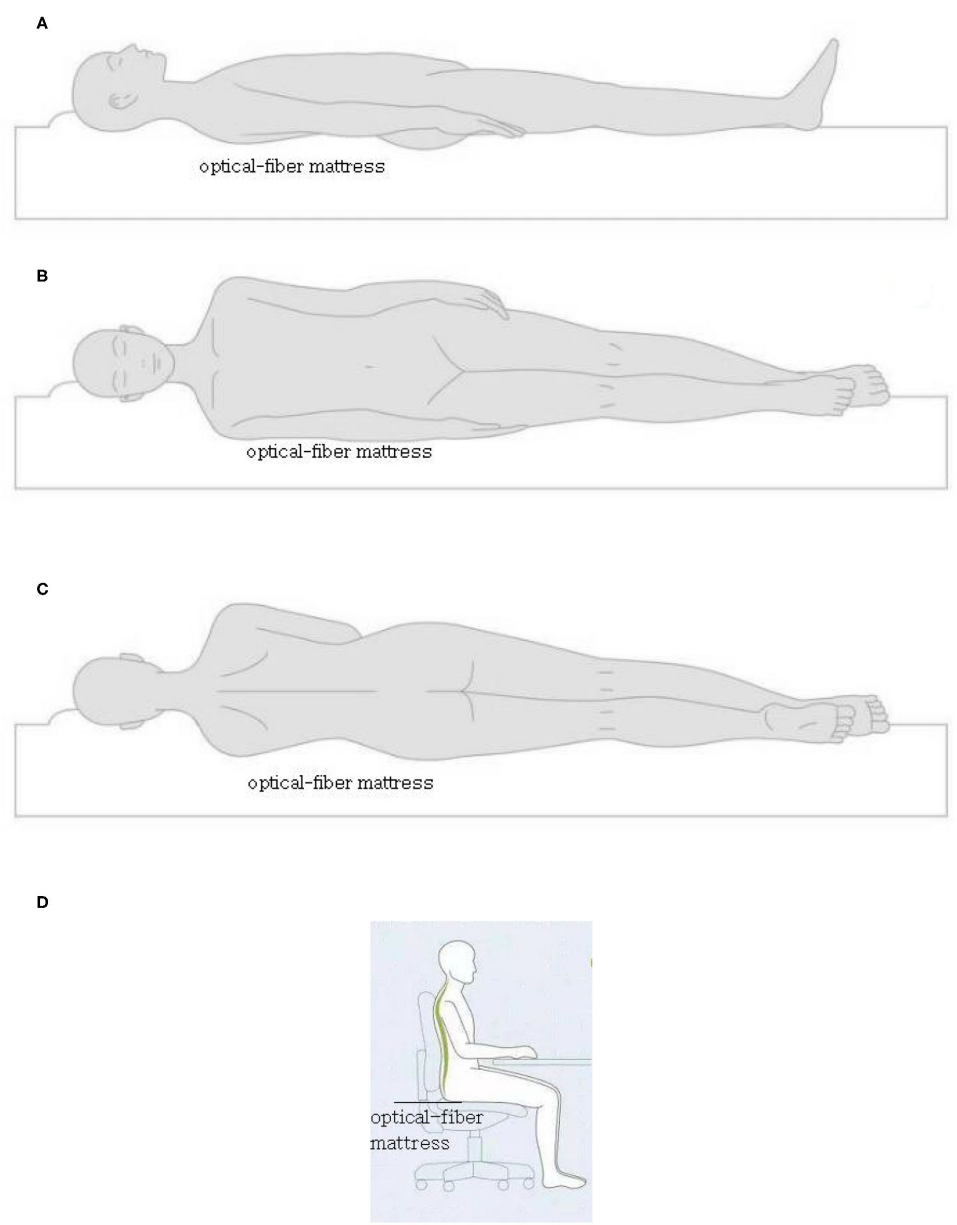
The four postures of the back **(A)** (near the chest), left **(B)**, right **(C)**, and sitting **(D)** (near the hips).

**Figure 13:**
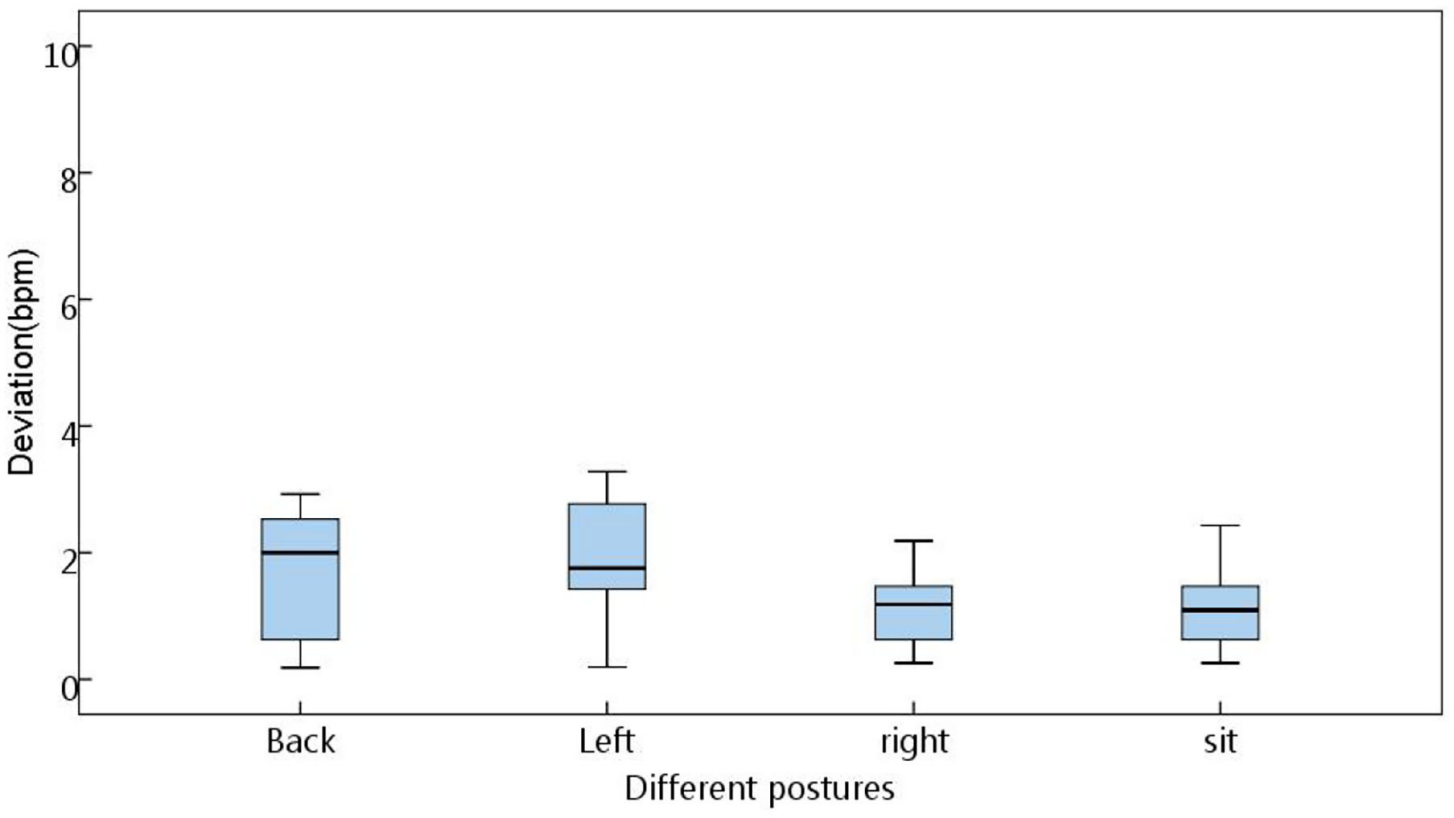
The deviation under four postures.

The four postures of the back (near the chest), left, right, and sitting (near the hips) (see Figure 12) were used to judge the range of errors under different postures. The results in the box diagram show that the states of right and sitting (near the hips) had better results, and the overall mean errors were within a measurable range (Figure 13). In order to assess statistical similarities, the paired sample test was conducted, of which Table 2 shows that the postures of back-sit, left-right, and left-sit were significantly different with *P* < 0.05.

**Table 2:** Paired sample test between two postures.

		**Mean value**	**Variance**	**Significance (P)**
Pail test	Back-left	−0.34464	1.26890	0.1104
Pail test	Back-right	0.36291	1.2637	0.102
Pail test	Back-sit*	0.54228	1.20925	0.0265
Pail test	Left-right*	0.70755	1.09075	0.004
Pail test	Left-sit*	0.88692	1.14747	0.001
Pail test	Right-sit	0.17937	0.84361	0.171

The meaning of the symbol * is that the postures of back-sit, left-right, and left-sit were significantly different with P < 0.05.

### Verification of Physical Movements

**Figure 14:**
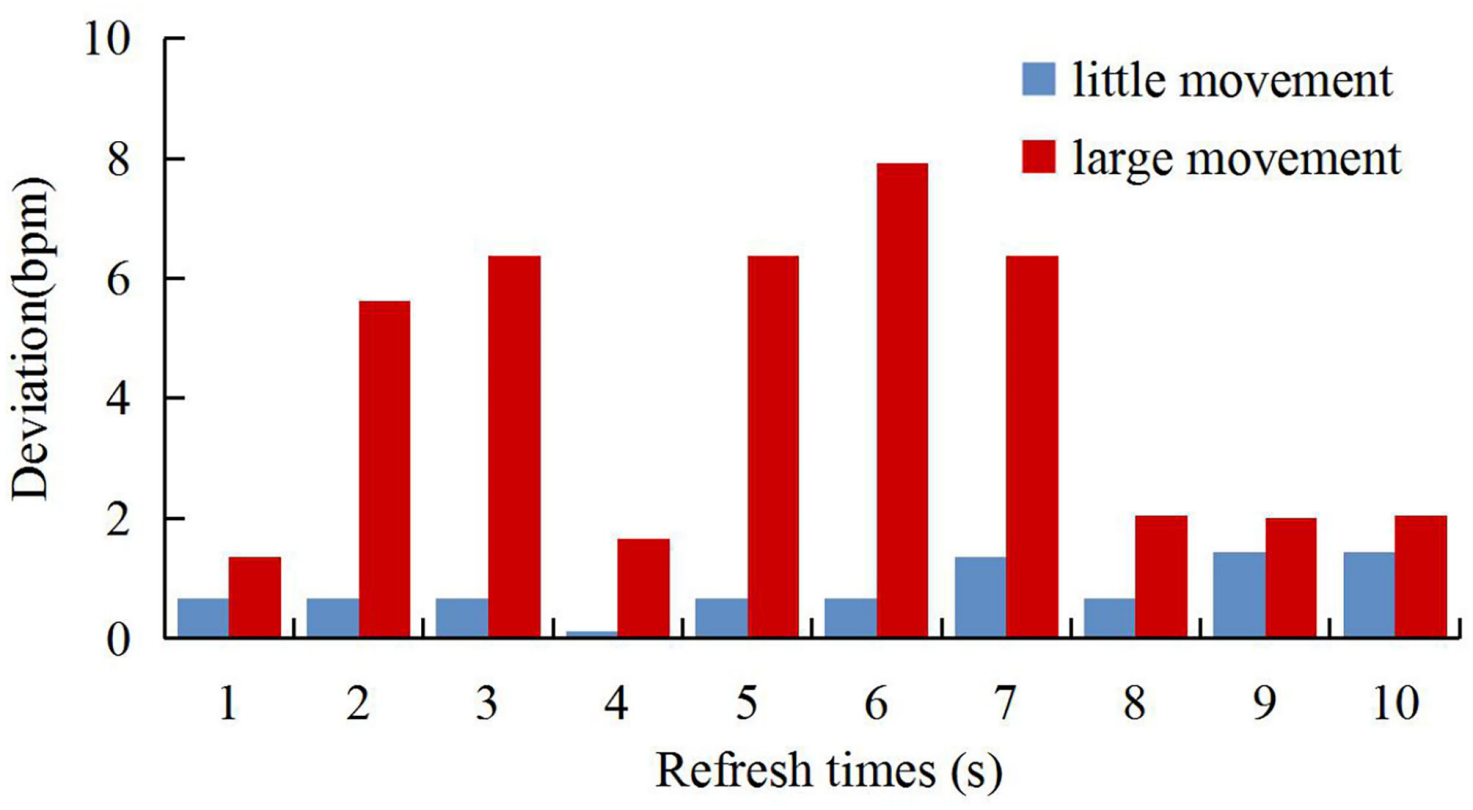
The deviation under physical movement.

Disturbed environments including small movements (such as movements of arms to see a mobile phone) and large movements (such as a big laugh) were simulated. The results are shown in Figure 14 where the abscissa represents updates of 10 heart rates in the moving state, and the ordinate represents deviations of heart rates between the system developed in this study and the standard ECG device. As can be seen from the figure, the algorithm of calculating the signal-to-noise ratio of the spectrum can determine the quality of the waveform. Under large movements, our system maintained the original values at low values of signal-to-noise ratios. However, the standard ECG had little impact, and large movements could affect heart rates. Therefore, the comparison between the two devices indicates a large deviation.

### Trend of the Heart Rate and Respiration Rate Changes Overnight

**Figure 15:**
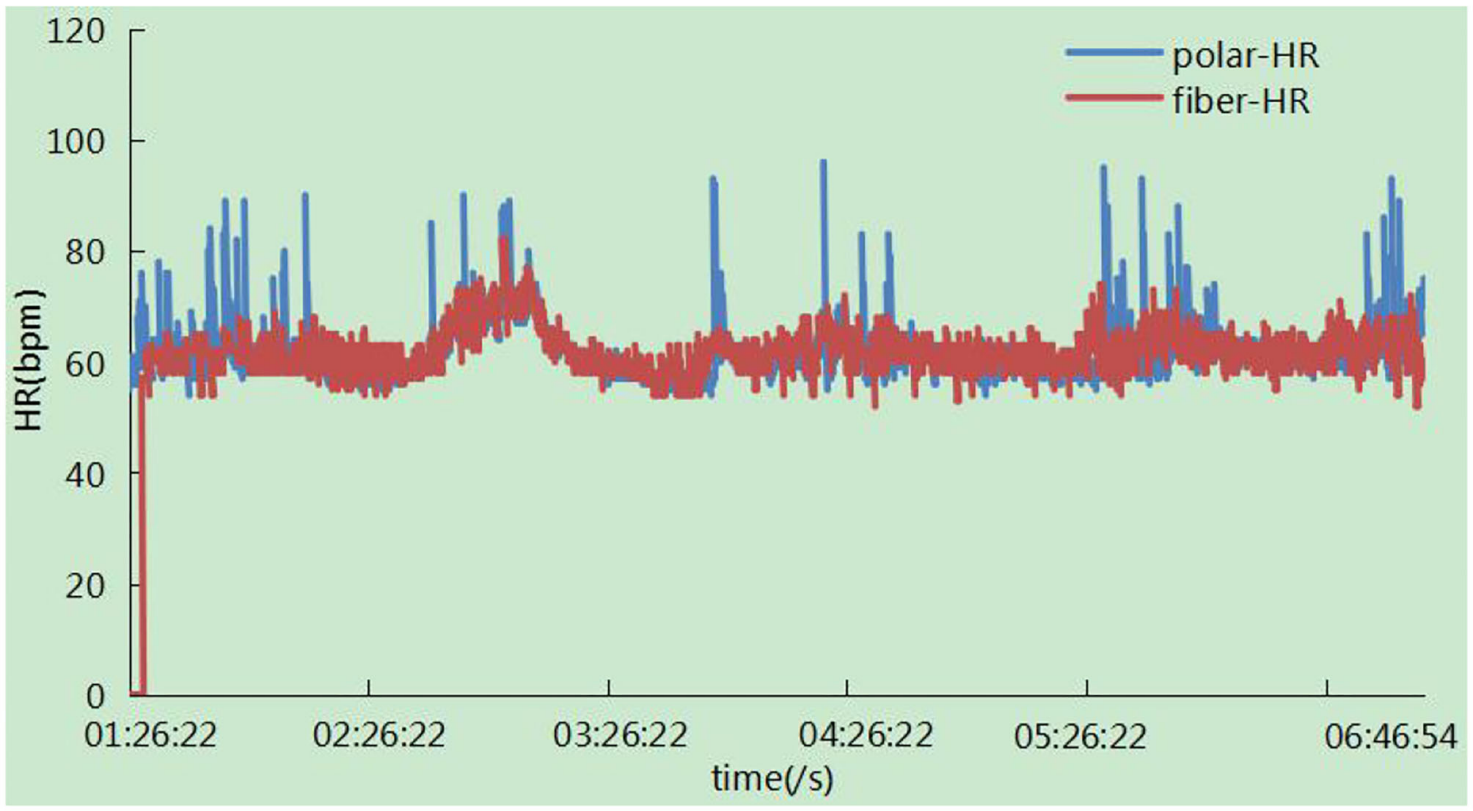
Represents the heart rate measurement of our system in one night.

**Figure 16:**
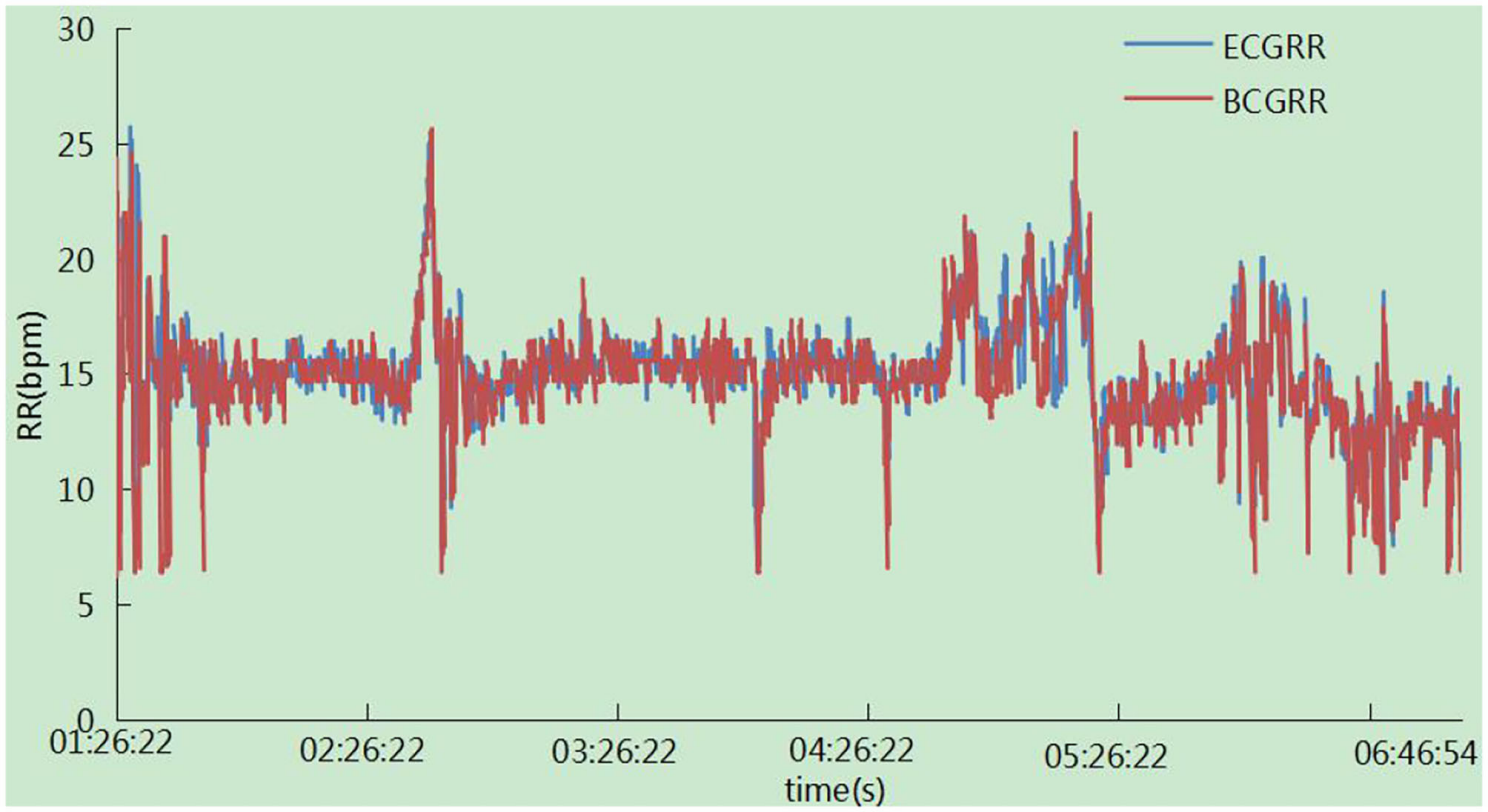
Represents the respiration rate measurement of our system in one night.

The trend of the heart rate is shown in Figure 15. There was a zero value around 01:26:22, which may result from the absence of the subject in bed. It has been observed that the trend of the two signals was highly consistent with each other, and there were some sudden changes in the polar device indicating large movements due to the high heart beats related to movements. In particular, signals were not calculated in this case in our system. The respiration rates were measured synchronously in one night by ECG-derived respiratory (EDR) measurements and our system. The result in Figure 16 showed that the trend of respiration rates had good correspondence.

### Validation of the Clinical Test

**Figure 17:**
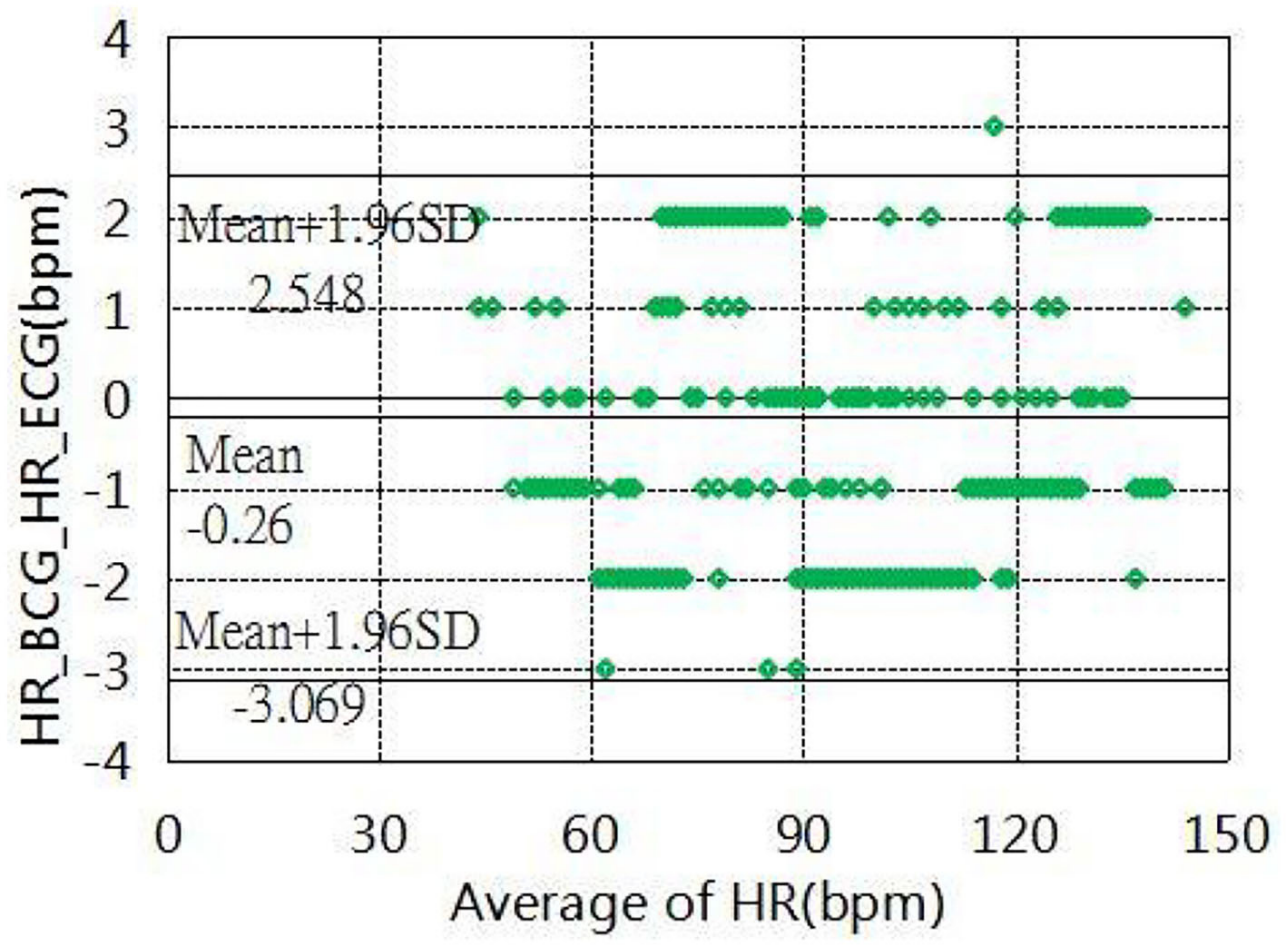
Bland–Altman plot of heart rate estimation.

**Figure 18:**
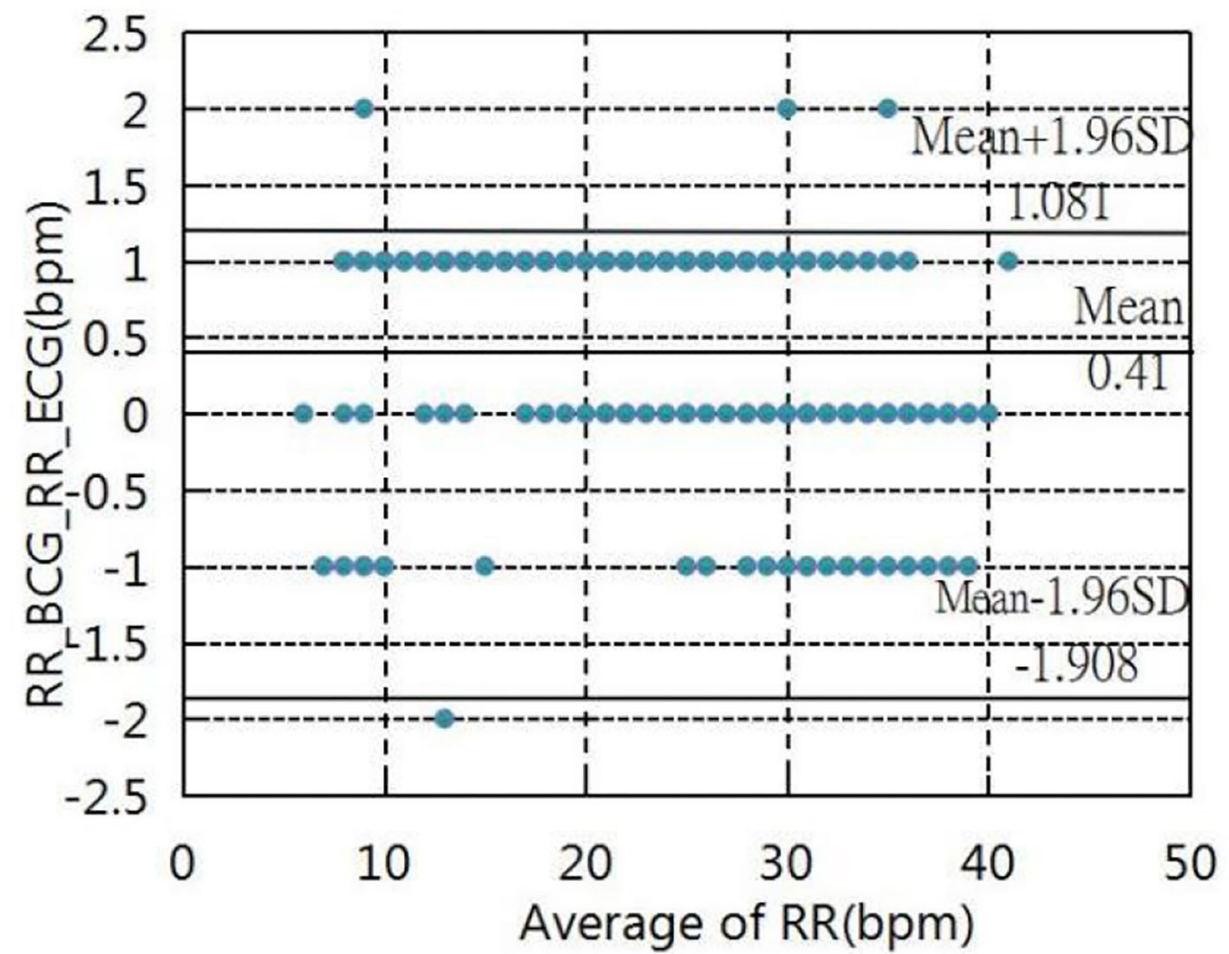
Bland–Altman plot of respiration rate.

The experiment was conducted in which the experimenter lay flat under resting conditions, simulating the human body as much as possible during sleep. System in the Navy General Hospital, Temple of Heaven medical. The heart and respiration rates were recorded for comparison with the Philips sure-signs VM6 medical monitor (USA) in several hospitals(Naval General Hospital and Temple of Heaven Hospital, Beijing, China). More specifically, the tests of heart rates were recorded with 215 groups of data measured for different individuals, which were analyzed and evaluated using the Bland-Altman plot (Figure 17). The results showed that the mean error in the range of a 40 to 145 heart rate within (±1.96 SD) was −0.26 ± 2.80 bpm, tests of the respiration rates were recorded by 167 groups of data measured at different intensities. The Bland-Altman plot (Figure 18) was used to analyze and evaluate the experimental results, which showed an average error of 0.41 ±1.49 bpm in the 6–40 respiration rates range.

### Discussion

It is shown that the method of adaptive regulations has good wide applicability for individuals of different weight. In the range of 40–80 kg, the output signal is limited to 1–2 V by adaptive adjustment. If the adaptive method is not used, the signal collected by the system will be too small to handle because the weight is too small, and will saturate because the weight is too much. Applying this method, a mattress embedded with an optical fiber can be used in individuals of different weights and different ages, so that the equipment has universal applicability.

For different postures, the measure errors had different situations. The states of right and sitting (near the hips) had better results, and the overall mean errors were within a measurable range. This indicates that the states of right and sitting are the best conditions for signal detection. This can guide other similar BCG signal acquisition equipment to obtain optimal detection conditions.

Under large movements, the signals collected by our system had a very low signal-to-noise ratio which was not as good as standard ECG equipment. Although the BCG equipment is not a substitute for the ECG equipment, it achieves most of the performance of the ECG equipment under certain conditions. However, in certain application fields, such as a strong electromagnetic interference environment and sleep state, the BCG equipment has a better application than ECG equipment. This later experimental verification also proves that BCG equipment has the same excellent detection performance as ECG equipment under sleep conditions and clinical tests.

## Conclusions

In this paper, a method of accurately estimating heart and respiration rates was designed under different actual conditions by the integration of an optical fiber sensor into a mattress. In this method, the adaptive feedback was provided to eliminate individual differences dynamically by automatically adjusting the driving voltage of the transmitter driving circuit and the magnification of the amplification circuit. Meanwhile, the statistical classification method based on spectral transformation was proposed in the detection of the perturbation state to find the fundamental and harmonic frequencies. Experiments were carried out in different individuals who had different breathing habits and used different postures. These experiments conclude the following results: (1) The algorithm can be used to detect the heart rate under weak perturbation states; (2) under different breathing patterns, the algorithm led to fluctuations which was within a suitable range; (3) the system can realize adaptive adjustments facing individual differences; (4) a large number of clinical trials showed that there was a high correlation between this equipment and the standard devices; and (5) this system could simultaneously extract the two physiological parameters (the heart rates and the respiration rates) by only using a single signal source. It is shown that the developed system using adaptive regulations and statistical classifications spectrum analysis had a good performance and it could easily be used under complex environments. Therefore, it can be concluded that this system in the paper has a broad application prospect as a non-contact sleeping mattress system in the field of healthcare (26–30). However, the measurement was not suitable for subjects who make large body movements and the experiments only tested healthy subjects. Therefore, future efforts will focus on (1) the extractions of heart rates with full resolutions under motion disturbances and (2) measuring various patients to establish a predictive relationship between measurement results and disease stages.

## Data Availability Statement

The raw data supporting the conclusions of this article will be made available by the authors, without undue reservation.

## Ethics Statement

Written informed consent was obtained from the individual(s) for the publication of any potentially identifiable images or data included in this article.

## Author Contributions

RZ, LD, ZZ, XC, JS, BC, ZM, and ZF collaborated on the research for various aspects of the paper. XC, ZZ, RZ, and ZF conceived and designed the experiments. RZ and JS performed the experiments and analyzed the data. ZF contributed reagents, materials, and analysis tools. RZ wrote the paper. LD revised the paper. All authors contributed to the article and approved the submitted version.

## Funding

This work was supported by the National Key Research and Development Project (2020YFC2003703, 2020YFC1512304, 2018YFC2001101, and 2018YFC2001802), the CAMS Innovation Fund for Medical Sciences (2019-I2M-5-019), and the National Natural Science Foundation of China (Grant 62071451).

## Conflict of Interest

RZ and JS were employed by Zhongke Guangrun Technology Co., Ltd. The remaining authors declare that the research was conducted in the absence of any commercial or financial relationships that could be construed as a potential conflict of interest.

## Publisher's Note

All claims expressed in this article are solely those of the authors and do not necessarily represent those of their affiliated organizations, or those of the publisher, the editors and the reviewers. Any product that may be evaluated in this article, or claim that may be made by its manufacturer, is not guaranteed or endorsed by the publisher.
